# Evidence-Based Phytoiatry, a New Approach in Crop Protection

**DOI:** 10.3390/ijms20010171

**Published:** 2019-01-05

**Authors:** Marcello Iriti, Elena Maria Varoni

**Affiliations:** 1 Dipartimento di Scienze Agrarie e Ambientali, Università degli Studi di Milano, via G. Celoria 2, 20133 Milan, Italy; 2 Dipartimento di Scienze Biomediche, Chirurgiche e Odontoiatriche, Università degli Studi di Milano, via Beldiletto 1/3, 20142 Milan, Italy; elena.varoni@unimi.it

**Keywords:** plant protection products, agrochemicals, sustainable crop protection, food security

## Abstract

In the past decades, the scientific quality of biomedical studies has been hierarchically depicted in the well-known pyramid of evidence-based medicine (EBM), with higher and higher levels of evidence moving from the base to the top. Such an approach is missing in the modern crop protection and, therefore, we introduce, for the first time, this novel concept of evidence-based phytoiatry in this field. This editorial is not a guideline on plant protection products (PPP) registration, but rather a scientific and technical support for researchers involved in the general area of plant pathology, providing them with evidence-based information useful to design critically new studies.

The term “evidence-based medicine” (EBM) was first introduced in 1992 in the *Journal of American Medical Association* (*JAMA*), referring to the use and the critical appraisal of the best scientific evidence to support clinical decision-making. This idea purported that all medical activities intended for diagnosis, prognosis and therapy should be based, not primarily on clinical experiences, but on solid scientific evidences coming from clinical research. Since then, EBM approach has continued to grow and spread, taking a pivotal place beside the competence of the clinician and the care preferences of the single patient.

This new way of thinking and acting now accounts the most updated evidence resulting from methodologically rigorous studies, mainly randomized controlled clinical trials (RCTs) and systematic reviews, which correspond to the highest level of scientific quality and represent the top of the EBM pyramid (Figure 1). The base consists of pre-clinical research (in vitro and in vivo experiments), expert opinions and case reports/series, while the middle part is composed of observational studies including case-control and cohort studies, and RCTs and systematic reviews constitute the top. RCTs are intervention studies considered the gold standard of clinical research to evaluate the efficacy/effectiveness of a therapy, because of their ability to minimize the biases over other types of clinical trials [1]. The cornerstone of RCT is randomization, which allows distributing each prognostic factor homogeneously between the test group and the control group. The tip of the pyramid, however, is occupied by systematic reviews, belonging to secondary literature. They are tools aimed at summarizing data coming from primary literature, such as RCTs, cohort and/or case-control studies. They aim at answering a well-focused question about etiology, diagnosis, therapy and prognosis of a certain disease, paying particular attention to the search methodology of studies to be included and providing a critical evaluation of the resulting quality of evidence. Systematic reviews are, whenever possible, associated with meta-analysis, which is a statistical analysis of all data coming from the different studies included; the latter provides a quantitative analysis of findings in support or against a certain treatment or risk factor in the contest of a specific disease. Underlying the importance of systematic review, the Cochrane Collaboration was founded in 1993, an international network with the specific aim of preparing and maintaining continuously updated and publically available systematic reviews on the impact of health interventions, which follow a unique standard of methodology.

In the EBM, the clinician is trained to identify the best scientific evidence currently available on a medical issue, after constructing an appropriate research question and using the reference database for medical literature (PubMed) for reviewing studies. To construct the clinical question, EBM proposes the PICO strategy, where P stands for Patients (with a particular condition or disease), I for the Intervention of interest (therapeutic, preventive, prognostic, diagnostic), C for Control or Comparison (the former defined as no intervention such as placebo, the latter as the standard intervention to be compared) and O for Outcome (the expected results coming from the intervention).

In the phytoiatric/phytopathological area, an evidence-based approach is still missing. Similar to the medical sciences, the efficacy of plant protection products can be assessed with diverse experimental designs each with different levels of scientific evidence. In general, active substances are initially assayed with in vitro tests evaluating their biostatic/biocidal activity on cultivable plant pathogens. To the next level, in planta experiments in controlled environments, i.e., phytotron cabinets, growth chambers, greenhouses and screenhouses, can be carried out to test the efficacy of both active substances and agrochemicals on model pathosystems as well as obligate pathogens. In these experimental conditions, phytotoxicity and adverse effects on non-target plant species can also be evaluated. Overall, in vitro and in planta experiments (pre-open field studies) can be compared to in vitro/in vivo preclinical research of biomedical area, at the base of the evidence-based phytoiatry pyramid (Figure 2). Following this parallelism, multi-year open field trials, with positive (untreated) and negative (reference standard) controls, performed in a completely randomized design are at the top of the pyramid, providing the highest level of evidence on efficacy of plant protection products including fungicides, elicitors, plant activators, insecticides and herbicides.

Of course, to design and perform an open-field trial is complex, and a number of general principles should be followed. First, the concept of Good Plant Protection Practice (GPP) was defined by the European and Mediterranean Plant Protection Organization (EPPO), a specific set of recommendations including guidelines on decision-making for choice of active substances and formulations, dosage (and appropriate volume), number of applications, timing or frequency of applications, equipment and method of application [2]. In addition, EPPO provides a number of standards covering two application fields: phytosanitary measures and plant protection products. Main topics include analysis of efficacy evaluation trials, efficacy evaluation of plant protection products (PPPs), number of efficacy trials, minimum effective dose, principles of acceptable efficacy and dose expression for PPPs, to name a few [3]. In addition, we have to take into account that legislations on PPPs are demanding, lengthy and costly, especially in the EU where issues related to their assessment and evaluation trials are controlled by the regulation EC 1107/2009 placed on the market of PPPs. During the approval process, the steps concerning the registration of new PPPs are declined because they require multi-year field trials to prove the efficacy of a given PPP against a target pest (sensu lato) under different pedo-climatic situations ruling out any negative effect on non-target organisms.

Not last, systematic reviews (and meta-analyses) are not used in phytoiatry/plant pathology research as a powerful tool to reach the highest level of evidence in a specific phytosanitary issue. Therefore, we believe that the plant protection sector can neither be apart from considering that different levels of scientific evidence actually exist nor disregard a modern evidence-based approach.

## Figures and Tables

**Figure 1:**
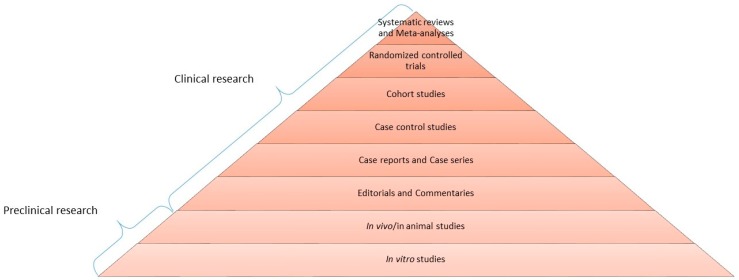
Pyramid of scientific evidence—Evidence-based medicine (EBM). In biomedical sciences, a pyramid represents the quality of scientific evidence; the base consists of pre-clinical research, with in vitro and in vivo experiments, which provides insights towards potential efficacy of a certain therapy. Clinical research represents a higher level of evidence, referring to in human studies; in this context, randomized controlled trials are methodologically the best current clinical studies. At the apex, updated systematic reviews and meta-analyses are considered the best available knowledge, suitable for clinicians to face decision-making (adapted from Varoni et al., 2015 [1]).

**Figure 2:**
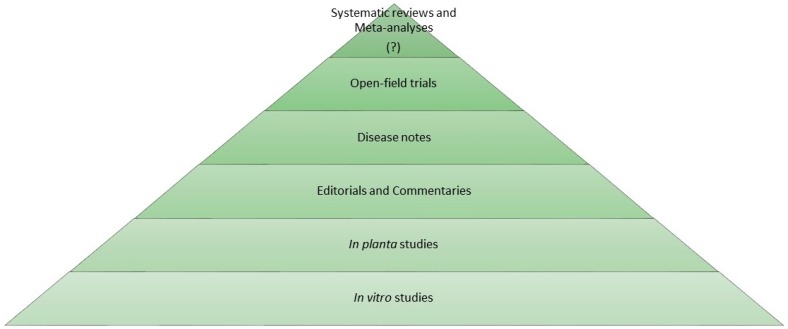
Pyramid of scientific evidence—Evidence-based phytoiatry (EBP). Suggested pyramid of the quality of scientific evidence in phytoiatry. In vitro and in planta experiments represent the level of the pre-open field research. Systematic reviews and meta-analyses need to be introduced in the phytoiatric/phytopathological area (‘?’ on the pyramid top).
